# Supplementary material for: Association between oral microbiome and seven types of cancers in East Asian population: a two-sample Mendelian randomization analysis
Source: Front Mol Biosci. 2023 Nov 21;10:1327893. doi: 10.3389/fmolb.2023.1327893 (PMC10702768; doi:10.3389/fmolb.2023.1327893)
Supplement: Supplementary file 1 [file DataSheet1.ZIP › Supplementary Materials/MR plots for tongue/tongue═╝/Prostate cancer/pheno.2467_to_prostate cancer_forest.pdf]

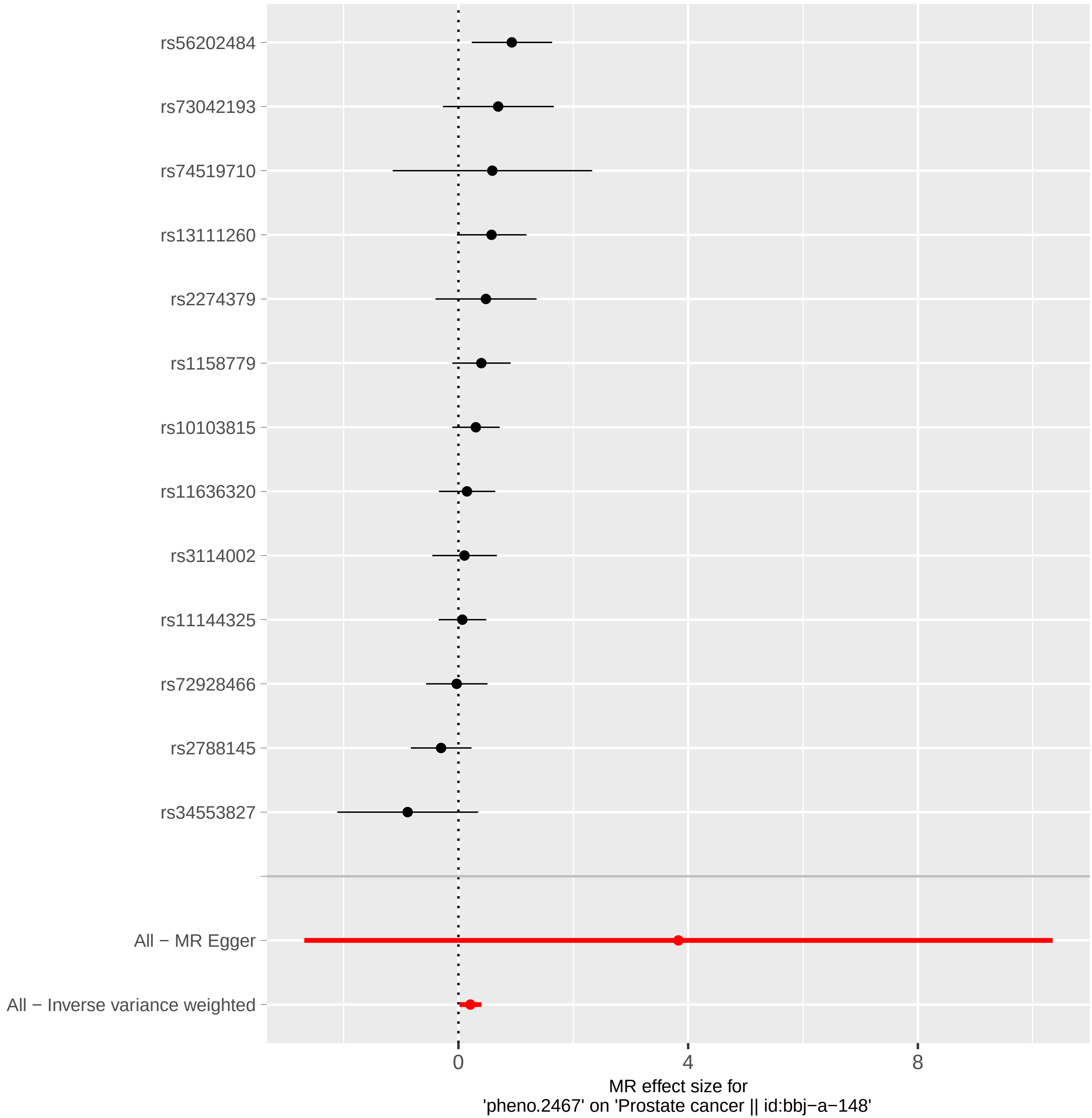

rs56202484
rs73042193
rs74519710
rs13111260
rs2274379
rs1158779
rs10103815
rs11636320
rs3114002
rs11144325
rs72928466
rs2788145
rs34553827
All − MR Egger
All − Inverse variance weighted
0
4
8
MR effect size for
'pheno.2467' on 'Prostate cancer || id:bbj−a−148'
